# Antimicrobial resistance pattern of anaerobic bacteria causing lower respiratory tract infections

**DOI:** 10.1186/s12866-023-03059-6

**Published:** 2023-10-23

**Authors:** Malini Shariff, Elizabeth Ramengmawi

**Affiliations:** Department of Microbiology, Vallabhbhai Patel Chest Institute, University of Delhi, Delhi, 110007 India

**Keywords:** Anaerobic bacteria, Respiratory Infections, Antimicrobial resistance

## Abstract

**Background:**

Anaerobes are normal flora of the human body. However, they can cause serious infections in humans. Anaerobic bacteria are known to cause respiratory infections like pneumonia and acute exacerbation of chronic lower airway infections. These are often missed due to the complexity of their isolation and identification. Hence, this study aimed to study anaerobes causing respiratory tract infections and determine their antibiotic susceptibility.

**Materials & methods:**

Clinical specimens such as bronchial aspirates and pleural aspirates collected from patients with respiratory diseases attending Vallabhbhai Patel Chest Institute were processed, the anaerobes isolated were identified, and their susceptibilities to various groups of antimicrobials were studied using standard microbiological methods.

**Results:**

Three hundred and fourteen patients were included in the study, 154 males and 160 females. Of these 314 patients, 148 (47%) yielded anaerobes in their clinical samples. Seventy patients had more than one type of anaerobic organism. Hence, 235 isolates were recovered belonging to as many as 17 genera. The MIC of seven antibiotics on 154 isolates was tested. The isolates belonged mostly to the genera *Bacteroides, Prevotella, Veillonella, and Actinomyces*. Variable resistance was observed to most classes of antibiotics by many genera.

**Conclusions:**

Metronidazole is commonly used against anaerobes, but the study showed that the isolates were 20–30% resistant to the antibiotic. Starting this as an empirical therapy might lead to treatment failure.

## Introduction

Anaerobic bacteria are an indigenous flora of the human body and are in abundance as compared to aerobic flora. Although they are normal flora, many of them behave as opportunistic pathogens and cause serious infections in humans. These include bacteraemia, brain abscess, subdural empyema, lung abscess, pneumonia, bronchiectasis, empyema, intra-abdominal infections, pelvic inflammatory disease, skin and soft tissue infections, diabetic foot infections, and osteomyelitis. Mixed infections along with aerobic infections are not rare, and it is important to identify the anaerobes along with the aerobes; otherwise, it will lead to treatment failure.

Anaerobic bacteria play a significant role in the prevention of oropharyngeal colonization by pathogens by competitive mechanisms. However, conditions such as smoking, alcohol abuse, poor oral hygiene, prolonged hospitalization, immunosuppression, chronic lung diseases, cancer, and cough reflex disorders can predispose a patient to infections in the respiratory tract, including bronchitis, lung abscess, thoracic empyema, or necrotic lung inflammation. Furthermore, antibiotic-related selection pressure on the oropharyngeal flora is another known risk factor for anaerobic respiratory tract infections. These organisms also play a significant role in chronic obstructive pulmonary disease (COPD). Aspiration of oropharyngeal secretions due to defects in the clearance mechanisms of the respiratory tract in COPD patients can further lead to these infections. [1–3].

Isolation of anaerobes in respiratory infections accounts for 8–16% [4], and are typically involved in aspiration pneumonia, necrotic pneumonia, lung abscess, pyothorax, and acute exacerbations of chronic lower airway infection. In acute bronchitis, anaerobes are only next to that of *Haemophilus influenzae*, α-haemolytic Streptococcus, and *Streptococcus pneumoniae*. They are detected in 93% of lung abscesses and 54% of pyothorax cases. Thus, anaerobic bacteria should not be neglected.

Several classes of antimicrobial agents have good activity against anaerobic bacteria, including penicillin alone or in combination with beta-lactamase inhibitors, cephalosporins, carbapenems, chloramphenicol, clindamycin, metronidazole, glycopeptides, macrolides, tetracyclines, and fluoroquinolones. However, the emergence of antimicrobial resistance of anaerobes over the past two decades has led to treatment failure. Beta-lactamase production is one of the most common resistance mechanisms [5, 6]. Approximately 20% of lung cancer patients’ isolates from bronchial secretions produced beta-lactamase. Amoxicillin/clavulanate was the only antimicrobial agent active against all the tested isolates [7]. Resistance to various classes of antibiotics has emerged in Gram-negative anaerobes across the globe, especially by *Bacteroides, Prevotella and Veillonella spp*. [8–10]. Furthermore, the Gram-positive anaerobes showed sensitivity to imipenem and cefoxitin, while the highest resistance was seen for metronidazole and penicillin G [5, 9, 11–14]. The rates of resistance may show variations among geographic areas and between countries and hence need to be tested for deciding on empirical therapy.

## Methodology

### Patients

Institutional human ethics clearance was obtained. Patients attending Vallabhbhai Patel Chest Institute for various respiratory tract problems, such as lung abscess, chronic obstructive pulmonary disease, asthma, and empyema, with acute exacerbations, as diagnosed by the clinicians using the ICD-10 document given by WHO, were included in the study. Patients with tuberculosis were excluded. Patients gave written informed consent for them to be included in the study.

### Clinical specimens

Bronchial aspirates (294), pleural fluid (4), BAL(14) and Endotracheal aspirates (2) were collected from patients with acute exacerbation with underlying respiratory illnesses who attended Vallabhbhai Patel Chest Institute. The underlying respiratory illnesses included chronic obstructive pulmonary disease (COPD)-36, interstitial lung disease (ILD)-84, malignancy-28, space-occupying lesion-9, and others-157. Samples were collected in plain sterile containers and anaerobic vacutainers (BD) and transported to the Microbiology Department for further processing. The specimens were processed within 2–3 h of receiving the samples.

### Isolation of anaerobes and aerobes

The samples were processed, both aerobically and anaerobically, and the isolates were identified by standard microbiological methods [15, 16]. Samples were cultured for anaerobes in the anaerobic cabinet (Bug Box, M/s Ruskin Technology Ltd/Bug box) and incubated in the same. Briefly, smears were prepared from the clinical samples and stained with Gram’s stain and were further inoculated into Robertson’s cooked meat medium (RCM), Brucella agar supplemented with vit K and hemin (BAP), kanamycin vancomycin laked blood (KVLB) agar and phenyl alcohol sheep blood agar (PEA). Plates and broth were incubated in anaerobic conditions at 37 °C for 72–96 h. They were examined after 48 h and at regular intervals until the appearance of growth. A subculture was performed from RCM after 48 h. into the above mentioned solid media plates and incubated as per the same conditions mentioned above. A standard loop of 0.01ml was used to inoculate the plates, and ≥ 10^3^ CFU /ml was considered significant. In BAL samples, since 100 ml of saline is already added, this is considered when calculating the concentration.

### Identification of the anaerobic isolate

The colonies were observed and further identified by standard methods given in the Wadsworth manual [16]. Briefly, Gram staining was performed on the smears from the colonies. Depending upon the Gram’s reaction and morphology, phenotypic tests were performed for further identification of the organisms. These included indole production, nitrate reduction, catalase production (15% H_2_O_2_), and sensitivity to vancomycin (5 µg), kanamycin (1000 µg), colistin (10 µg) discs, and 20% bile. For Gram-positive bacteria, in addition to the above, sensitivity to sodium polyanethol sulfonate (SPS) was also determined. Identification was further confirmed by automated methods using Vitek 2 anaerobic identification cards (Cat # ANC 21,347, BioMerieux) and/or MALDI-TOFF (Bruker, Daltonics, Germany).

### Antibiotic susceptibility of anaerobic clinical isolates to various classes of antimicrobial agents

The minimal inhibitory concentrations (MICs) for penicillin G, cefoxitin, tetracycline, chloramphenicol, moxifloxacin, clindamycin, and metronidazole were determined according to Clinical Laboratory Standard Institute (CLSI) recommendations [17]. CLSI recommends the agar dilution technique for MIC determination. However, only for *Bacteroides*, micro broth dilution has been recommended. Hence, we also used micro broth dilution for our *Bacteroides* isolates and agar dilution for all the other isolates. The results were interpreted as per the CLSI guidelines. The results of penicillin for Gram-negative organisms were interpreted as per EUCAST since it is not given in CLSI guidelines. *Bacteroides* is inherently resistant to penicillin and hence were not tested.

### Isolation and identification of aerobes and facultative anaerobes

According to standard microbiological methods, clinical specimens were processed for aerobes and facultative anaerobes [15]. Briefly, specimens were inoculated on blood, chocolate, and MacConkey agar. A staph streak was made in blood agar plates to look for satellitism shown by *H. influenzae*. Plates were incubated at 37 °C overnight. In addition, Blood agar plates were incubated in 5–10% CO_2_. Isolated organisms were identified by standard biochemical tests, and antibiotic sensitivity was tested by Kirby Bauer’s method and interpreted according to CLSI guidelines [18]. X, V, or XV factors were required to identify *H. influenzae*. 

## Results

Three hundred and fourteen patients were included in the study: 160 males and 154 females. Out of these 314, 148 (47%) patients yielded anaerobes either as pure (both mono and polymicrobial) or along with aerobes in their clinical samples. Seventy-eight patients had one type of anaerobic organism, 55 had two types 14 had 3 types and one had five types.

A total of 235 anaerobic isolates belonging to 17 genera were isolated from these 148 patients. The organisms isolated are given in Table 1.

**Table 1:** List of anaerobic Organisms isolated in the study (n = 235)

Name of Organisms	Number of isolates
Gram Positive Cocci	
Parvimonas micra (Micromonas micros*)	29
Peptoniphilus assacharolyticus (Peptostreptococcus assacharolyticus*)	10
*Peptostreptococcus anaerobius*	12
*Anaerococcus prevotii*	5
*Fingoldia magna*	2
**Gram Positive Bacilli**	
*Actinomyces*	54
*Propionebacterium acnes*	3
*Bifidobacterium species*	8
Terrisporobacter glycolicus*(Clostridium glycolicus)	1
*Atopobium parvulum*	1
*Gamella species*	2
*Eubacterium species*	1
**Gram Negative Cocci**	
*Veillonella species*	44
**Gram Negative Bacilli**	
*Prevotella species*	49
*Bacteroides species*	11
*Fusobacterium*	3

*- old nomenclature

*Actinomyces* (54) was the most common, followed by *Prevotella spp.* (49) and *Veillonella spp.* (44). These are known to cause lung infections. *Terrisporobacter glycolicus* (previously *Clostridium glycolicus*) is not a known pathogen but has been implicated in respiratory infection but with low prevalence. However, it is not a normal flora of the upper respiratory tract. A few of these are also normal flora. In 45 isolates (anaerobes-30 & aerobes-15), both VITEK and MALDI-TOFF were performed, and the results were congruent.

One hundred and forty-five patients yielded aerobes, out of which 76 were along with anaerobes. One hundred and eighty-three isolates were obtained from 145 patients on aerobic culture. From which 107 samples had one type and thirty-eight samples had two types of aerobic organisms.

Aerobic organisms isolated are given in Table 2.

**Table 2:** List of Aerobic organisms isolated in the study. (n = 183)

Name of Organisms	Number of Isolates
**Gram Positive Cocci**	
*Staphylococcus epidermidis*	14
*Staphylococcus aureus*	02
*Staphylococcus hominis*	1
*Staphylococcus lentus*	2
*Streptococcus Sps*	105
*Streptococcus pneumoniae*	14
*Enterococcus Sps*	02
**Gram Positive Bacilli**	
*Lactobacillus Sps*	19
*Capnocytophaga sputigena*	3
**Gram Negative Bacilli**	
*Klebsiella pneumoniae*	06
*Escherichia coli*	04
*Escherichia fergusonii*	01
*Pseudomonas aeruginosa*	03
*Pseudomonas species*	03
*Salmonella enterica*	01
*Enterobacter species*	02
*Acinetobacter sps.*	01

The majority of these were commensals from the oral flora. However, some of these have been implicated in lower respiratory infections. *Klebsiella pneumoniae* (6), *Escherichia coli* (4), *Pseudomonas sp.* (6), and *Streptococcus pneumoniae* (14) were the known pathogens.

After standardizing the methodology, MIC was determined for seven antibiotics on 154 anaerobic isolates. These isolates belonged to the *genera Bacteroides, Prevotella, Veillonella, and Actinomyces*. Sensitivity to penicillin ranged from 19 to 24% in Gram-negative bacteria and 6% and 20% in *Parvimonas micra* and *Actinomyces*, respectively. A total of 83–100% of isolates were sensitive to cefoxitin. Similarly, most strains showed good sensitivity to tetracycline and chloramphenicol. A total of 75 to 82% were sensitive to metronidazole, but some genera, such as *Propionebacterium acnes* (33%), *Peptoniphilus assachrolyticus* (33%) and *Parvimonas* (46%), showed lower susceptibility. Surprisingly, considerable resistance was seen for moxifloxacin (4–30%) and clindamycin (12–60%). The sensitivity varied among different genera. Some pathogens, such as *Atopobium parvulum*, *Propionibacterium acnes*, *Terrisporobacter glycolicus*, and *Fingoldia magna*, were 100% resistant to penicillin. However, since the number of isolates was few, this cannot be commented upon. (Table 3)

**Table 3:** Resistant pattern of anaerobes to various antibiotics by MIC = N (%)

Anaerobic Organisms	No. of isolates tested (n = 154)	Penicillin	Cefoxitin	Tetracycline	Moxifloxacin	Clindamycin	Chloramphenicol	Metronidazole	Meropenem
Resistant breakpoints (µg/ml		≥ 2	≥ 64	≥ 16	≥ 8	≥ 8	≥ 32	≥ 32	≥ 16
Gram Negative Anaerobes									
*Bacteroides Sps.*	10		0(0)	0(0)	3(30)	6(60)	0(0)	2(20)	0(0)
*Prevotella Sps*	31	20(65)	4(13)	929)	3(10)	5(15)	2(6.5)	10(32)	0(0)
*Veillonella Sps*	33	19(58)	3 (9%)	6(18)	4(12)	4(12)	7(21)	6(18)	0(0)
Gram Positive Anaerobes									
*Actinomyces*	45	29(64)	1	8(18)	2(4)	7(16)	3(6.7)	-#	0(0)
*Atopobium*	1	1(100)	0	0(0)	1(100)	0(0)	0(0)	0(0)	0(0)
*Bifidobacterium Sps*	7	4(57)	1(14)	1(14)			0(0)	3(42)	
*Propionebacterium acnes*	3	3(100)	0	0(0)		0(0)	0(0)	-#	0(0)
*Terrisporobacter glycolicus*	1	1(100)	0	0(0)			0(0)	1(100)	
*Gamella Sps*	2	2(100)	0	1(50)			1(50)	1(50)	
*Parvimonas micra*	15	11(73)	1(6)	2(13)	2(13)	2(13)	2(13)	5(33)	0(0)
*Anarococcus prevotii*	1	0(0)	0	0(0)	1(100)	0(0)	1(100)	0(0)	0(0)
*Peptoniphilus assachrolyticus*	3	2(67)	0	1(33)	0(0)	0(0)	0(0)	2(67)	0(0)
*Peptostreptococcus anaerobius*	1	0(0)	0	0(0)		0(0)	0(0)	0(0)	
*Fingoldia magna*	1	1(100)	0	0(0)	0(0)	0(0)	0(0)	0(0)	0(0)

# Not done due to resistance of the genus to Metronidazole

## Discussion

Anaerobes are present as normal flora of the human body. They cause infections in different parts of the body, including the respiratory tract. Due to cumbersome procedures, anaerobic bacteria often remain unidentified in clinical practice. Thus, there is a paucity of data on anaerobes causing respiratory infections. Empirical therapy in most cases is beta-lactam, metronidazole or clindamycin [19, 20]. Recently, antibiotic resistance in anaerobes has been observed. Therefore, empirical therapy with known antibiotics may not be effective in most cases.

In the current scenario, anaerobes play an important role in diseases, especially respiratory infections. *Pepto streptococcus*, *Prevotella*, *Veillonella*, and *Parvula* are commonly isolated from respiratory infections. Polymicrobial anaerobes and/or mixed infections with aerobes, especially in acute exacerbations of chronic lower respiratory tract infections are a common feature. Anaerobes are seen in most cases of lung abscesses and pneumonia. In our study, 47% had anaerobes, of which poly-microbial infections were seen in 70% of clinical samples. Among the 145 samples positive for aerobes a total of 76 (52%) samples showed both aerobes and anaerobes. This is in concordance with many other studies where 68% of infections were poly-microbial [21, 22].

In the Indian scenario, a study by De et al., the isolation of both aerobes and anaerobes from 100 patients with pleuropulmonary infections resulted in anaerobes alone being recovered in 14% of the patients, while 58% of cases showed a mixture of both. Anaerobes were recovered in 65.6% and 68.4% of samples from Empyema and Pleural effusion, respectively; anaerobic bacilli predominated (*Prevotella melaninogenicus*, *Fusobacterium spp*., *Bacteroides spp*.), followed by Gram-positive anaerobic cocci (*Pepto streptococcus spp*.) [23]. Other studies also support this observation [24, 25].

There are a few Indian studies on chronic pneumonitis caused by *Fusobacterium*, *Veillonella*, and *Prevotella (Bacillus) melanogenicus* [26, 27].

Beta-lactam antibiotics are the drug of choice in the treatment of anaerobic infections. They have a broad spectrum of activity with low toxicity and are efficacious across anaerobic genera. However, recent resistance to this has been observed with a prevalence of 60–80% in European countries [11, 12, 28], USA (65%), Canada 63.5% and as high as 91% in Korea [8–10]. In our study, the resistant rates ranged from 58 to 65% in Gram-negative anaerobes and as high as 100% in Gram-positives. Though in most studies they were found to be susceptible [9, 12, 13], our number of isolates was from 7 to 15 and hence we cannot comment on it.

Recently, resistance to metronidazole has emerged. Bacteroides species showed a resistance of 15%, in Western countries [29], and up to 30% in a few Asian regions [30, 31]. In India, metronidazole resistance varied from 7 to 52% [32, 33]. In our study, 20% resistance was observed. Metronidazole resistance is also emerging in other genera, namely, *Prevotella* and *Veillonella* [34]. In our study, 32% and 18% were observed in *Prevotella* and *Veillonella*, respectively. Resistance as high as 28% was seen in Gram-positive anaerobes [35, 36]. In India, only 6.8% were resistant to metronidazole [32]. In our study, 42% of *Bifidobacterium* were resistant, but due to the smaller number of isolates (7), this cannot be commented upon.

The emergence of metronidazole resistance mandated the use of Carbapenems for the treatment of anaerobes. Carbapenem resistance has been observed to range from 1 to 9.6% in Western countries. East Asian literature shows 9–15% imipenem resistance across species [14]. In India, 0.6% resistance was seen. In Pakistan, 24.1% imipenem resistance was observed in metronidazole-resistant strains [37]. However, all our isolates were sensitive to meropenem. This could be because we used meropenem instead of imipenem, which was used in many earlier studies.

Resistance to cefoxitin is also on the rise. 17.2% and 35.3% were observed [32, 38]. A higher resistance, 48.2%, was observed in *Bacteroides* species [32]. However, in the present study, resistance in only 12% of *Prevotella* and 9% of *Veillonella* was observed. Among Gram-positive anaerobes, 4% and 6% resistance of *Bifidobacterium* and *Parvimonas*, respectively, was observed. All isolates of *Bacteroides* were sensitive to cefoxitin.

Due to its broad spectrum of activity, clindamycin has been used in non-severe anaerobic infections. However, over the last twenty years, resistance to clindamycin has increased by 32.4% worldwide, as seen in the study by Gajdacs et al., 2017 [20)]. In India, overall resistance was 42.6% higher in *Bacteroides* (53.6%). *Bacteroides fragilis* showed a resistance of 46.8% [32], which is like many other studies [38, 39]. In our study, 20–69% resistance was observed in Gram-negative anaerobes, with a higher percentage in *Bacteroides sp.* (60%) and up to 16% in Gram-positive anaerobes. This is like that in Asian countries [40]. Resistance of *Prevotella sp.* to Clindamycin was 30–40% in European countries, and 45–62% in Asian countries [8, 41].

All our isolates were sensitive to chloramphenicol, but more than half were clustering around the breakpoints of 8 − 4 µg/L. A case of MIC creep over time may pose a threat [32]. However, in our study, resistance to chloramphenicol was seen in *Veillonella* (21%), *Prevotella* (6.5%), and *Parvimonas* (13%), with breakpoints of 32 µg/L. This is surprising, as chloramphenicol is not commonly used in our hospital due to its toxicity.

Moxifloxacin, a fluoroquinolone is one antibiotic that demonstrated activity against anaerobes. However, resistance has been observed in *Bacteroides* and *Clostridium Spp.* It is between 30 and 42% [8, 20, 42]. *Fusobacterium* and *Prevotella Spp.* have also shown varied resistance across studies. This is due to different studies that have used either EUCAST or CLSI guidelines which have different breakpoints. In our study, we observed only 3% resistance in *Bacteroides sp*. and 10–12% in *Prevotella* and *Veillonella spp*. This could be because we do not use moxifloxacin routinely in our hospital even for aerobic infections.

There are a few limitations to the study. All our isolates were respiratory. We did not look for resistance genes in the isolates, and some of the genera had fewer isolates and thus cannot be generalized for that genus.

## Conclusion

To conclude, as observed in our study, metronidazole when used as a monotherapy, will not be effective as an empirical therapy. A combination of Carbapenem along with clindamycin should be the drug of choice to start as an empirical therapy in patients with clinical suspicion of anaerobic infections. Since most respiratory infections are polymicrobial with both Gram-positive and Gram-negative organisms, these will cover both.

## Data Availability

The data used are available from the corresponding author.
